# A New Principle for Measuring the Average Relative Humidity in Large Volumes of Non-Homogenous Gas

**DOI:** 10.3390/s19235073

**Published:** 2019-11-20

**Authors:** Detlef Lazik, Gerrit H. de Rooij, Walter Lazik, Ralph Meissner

**Affiliations:** 1 Helmholtz Centre for Environmental Research—UFZ, Theodor-Lieser-Strasse 4, 06120 Halle (Saale), Germany; gerrit.derooij@ufz.de (G.H.d.R.); walterlazik50@gmail.com (W.L.); 2 Helmholtz Centre for Environmental Research—UFZ, Lysimeter Station, Dorfstrasse 55, 39615 Falkenberg, Germany; ralph.meissner@ufz.de

**Keywords:** relative humidity, water vapor pressure, sensor, vapor-selective membrane, tubular form factor, representative measurement, heterogeneous systems

## Abstract

Due to the current extent of arid regions, the pressure on available water resources is increasing. A suitable measure for water availability and dynamics in dry soil is the relative humidity of the soil air. Due to the heterogeneity of soil, water inputs, and root water uptake, the humidity of soil air will vary in space. Therefore, area-representative measurement methods are needed to find a representative measure of the soil water status. Existing sensors for the direct determination of relative humidity only represent a single location with a spatial extent of up to several cm. We introduce a new measuring principle that averages over a spatially heterogeneously distributed relative humidity. It is based on the selective diffusion of water vapor pressure through a tubular semipermeable membrane to/from a closed measurement chamber. A measured pressure change is sensitive to the water vapor pressure and enables, without any external calibration, to estimate an average of the relative humidity. The comparison of our first laboratory prototype of the new sensor with calibrated reference sensors for relative humidity in a range of approx. 4 to 100% proves the linearity of the measuring method and its high accuracy. For further optimization improved reference measurement techniques are necessary. A potential application is the improvement of water use efficiency in irrigated agriculture.

## 1. Introduction

Traditionally, the study of the dynamics of soil water focuses on the liquid water phase. During periods when the soil is moist this is generally considered justified, because the contribution of water movement in the gas phase is assumed to be much smaller than that of liquid water flow. During dry periods, the capillary connection between the soil surface and the deeper subsoil is lost. When that is the case, water movement in the vapor phase contributes significantly to the soil water balance, although estimates of the magnitude in this term vary widely [1,2,3,4,5,6,7,8,9]. Driven in part by the global expansion of drylands [10], interest in water movement in dry soils is increasing, as evidenced by an increasing focus on the dry end of soil water retention curve ([10] and references therein) and the inclusion of water vapor fluxes in numerical models for the unsaturated zone [11]. However, our capability to measure the soil water vapor status in the soil lags behind our ability to measure the content of liquid water and its matric potential (describing its capillary tension), as is clearly apparent from the results of Dane and Topp [12]. This standard work for methods of soil analysis lists nine fundamentally different methods to measure the soil water content, and ten different methods to measure the potential of soil water (the soil water potential is a measure for the work required to extract an infinitesimal amount of water from the soil) but does not describe a single method to measure the water vapor pressure or soil air humidity in situ. Up to now, only buried mm to cm -scale air humidity sensors seem to have been applied in the soil, e.g., [13].

In general, there is a large range of measuring principles for determining relative humidity. The current broad state of development is illustrated in standard works, e.g., [14,15,16] and reviews [17,18,19,20,21,22,23,24,25]. In particular, current developments of fiber-optic methods show not only its applicability under harsh environmental conditions, as expected e.g., in soil, but also a larger spatial support. For example, in [25] fiber-optic sensors with a sensitive length of up to 12 cm are described.

Recently, a method to measure the concentration of CO_2_ in the soil using robust, long-term stable, tubular gas selective membranes buried at desired depths was demonstrated. The method has the advantage of providing an average of fluctuating local concentrations within the dynamically changing, heterogeneous multi-phase system soil over an area in the order of $10^{0}$ to $10^{3}$ m^2^ [26,27,28]. The combination of a self-calibrating sensor design [29] with a specially constructed water vapor compensating membrane [30] allowed the determination of small CO_2_ concentrations due to soil respiration in face of a temperature-dependent varying water vapor pressure in humid soils. Such membrane-based sensors can be used to analyze various gases as shown by computer simulations [31] and experiments, e.g., [32].

This paper introduces a new principle for direct measurement of relative humidity RH [%] that enables a scale-dependent measurement based on a conceptually new tubular membrane-based sensor design. This membrane-based humidity sensor (MHS) in principle can handle the same scales as the CO_2_ sensor. Particularly for sensors with tube lengths of several meters it is possible that the temperature along the sensor will not be uniform. The saturated vapor pressure of water varies considerably with temperature, which impacts local *RH* values to which the sensor is exposed and which it needs to average. To avoid an additionally effort due to the measurement of local temperatures, it makes it paramount that the measurement principle is such that the dependence on such temperature variations is as small as possible.

The MHS is based on the water vapor diffusion through its membrane, which is driven by the water vapor pressures on both faces of the membrane. Because diffusion is the mechanism upon which the sensor is based, its response function is expected to be highly linear assuming the membrane diffusivity is independent of the water vapor concentration. This linearity creates the potential to develop sensors that integrate over a spatio-temporal highly heterogeneous vapor pressure distribution in order to generate robust measurement values representative for tube lengths with orders of magnitude ranging from $10^{-1}$ to $10^{1}$ m. One such application would be a sensor with membrane tubes of several meters that can be buried in the soil. As shown in [13], the RH readings can be converted to a water potential. If the sensor is buried in the root zone of an irrigated agricultural field, it can help schedule irrigations to maintain the water potential in the root zone within a range that maximizes the crop yield per volume of irrigation water. If the sensor is buried below the root zone in natural land, it may contribute to improved estimates of groundwater recharge. In case large-scale data are needed that can realistically only be acquired by remote sensing, such data will probably require calibration with ground-truth data, which our technology can deliver with a much larger footprint than typical mm to cm -scale humidity sensors that measure the humidity within a measurement chamber with a volume below 1 cm^3^ that is in contact with a poorly defined but tiny soil volume.

## 2. Materials and Methods

### 2.1. Measurement Principle and Theoretical Basis

The MHS consists of a tubular non-porous symmetric membrane which is permeable to gas. The inside of the tube can be considered as the measurement chamber, which can be closed on both ends by valves. Together with the membrane it comprises the measurement cell of the MHS.

If the composition of the gas of interest (outside the cell) differs from that gas inside the measurement chamber, the partial pressures of the various components of the gas there will change by permeation through the membrane. The operation principle of an MHS is based on these changes.

For a given partial pressure difference the permeation depends on the material-dependent permeability coefficient $P_{j}=S_{j}D_{j}$ of a gaseous compound *j* ($D_{j}$ (m^2^/s)—diffusion coefficient, $S_{j}$ (mol/mol)—solubility), which can be expressed in (m^2^/s) assuming Henry’s law for gas solution to be valid (dimensionless solubility $S_{j}$ equal to the inverse dimensionless Henry constant [33]). The permeability of a gas component *j* can be related to that of a second gas component *k* defining its selectivity $f_{jk}=P_{j}/P_{k}$. Table 1 shows the permeability of gas components of air for a silicone rubber from Robb [34] (converted) and gas selectivity coefficients $f_{jw}$ with respect to the gas component of interest water vapor (index *w*).

In order to carry out a measurement, the measurement chamber is flushed with a gas of known composition long enough to establish steady-state molecular fluxes within the membrane (conditioning step). By closing the measurement chamber at time *t* = *t*_0_, the consecutive measurement step is started. The change of the number of gas molecules within the chamber can be expressed as a pressure change according to the ideal gas law and Dalton’s law of partial pressure. Early in the measurement step, the gas composition in the measurement chamber deviates little from the initial condition established in the conditioning step. Up to this time, called *t_lin_* (s), the pressure changes linearly with time and can then be described by the approximate solution [33]:

(1) $${\frac{dp}{dt}|}_{t\to to}=\alpha =p^{o}gP_{k}\sum_{j}f_{jk}(\chi_{j}^{o}-\beta \chi_{j}^{i})$$

The superscripts “*o*” and “*i*” define parameters outside and within the measurement cell. $\chi_{j}^{i},\;\chi_{j}^{o}$ are the mole fractions of gas component *j* within the measurement chamber and outside, and $\beta =p^{i}/p^{o}$ is the ratio of the inside $p^{i}$ (Pa) and outside $p^{o}$ (Pa) gas pressures.

The geometry factor g (m^−2^) accounts for the cylindrical shape of the gas-selective cell (gas volume V (m^3^), length L (m), outer $R_{o}$ (m) and inner $R_{i}$ (m) radius of the gas selective tube):

(2) $$g=2\pi \;L{\left(V\ln R_{o}/R_{i}\right)}^{-1}$$

The composition of the outer gas is arbitrary. To highlight the contribution of the single gas component *k* to α, we consider two cases that only differ in the molar composition $\chi_{k}^{o}$ of that component. A difference $\Delta \chi_{k}^{}=\chi_{k}^{o}-\chi_{0k}^{o}$ results in a dilution of the other gas components by the factor $1-\Delta \chi_{k}^{}$, where $\chi_{0k}^{o}$ is the molar fraction of this gas in a reference state. We are now able to reformulate Equation (1) to indicate the contribution of $\Delta \chi_{k}^{}$ by:

(3) $$\alpha =\frac{p^{o}}{\tau_{k}}\sum_{j}f_{jk}(\delta_{jk}\Delta \chi_{k}^{}+(1-\Delta \chi_{k}^{})\chi_{0j}^{o}-\beta \chi_{j}^{i})$$

where $\delta_{jk}$ is the Kronecker delta and $\tau_{k}={\left(gP_{k}\right)}^{-1}$ the gas-specific cell constant of the measurement chamber for the gas component *k*.

In order to determine the mole fraction of water vapor $\chi_{w}^{x}$ of ambient air (denoted *x*), we expose one of two identical measurement chambers to this air, and the other to drier air with $\chi_{w}^{d}$, while both are purged by the same gas (e.g., air from a pump) during conditioning. Considering $P_{k}\to P_{w}$ and $f_{jk}\to f_{jw}$ in Equation (3) the difference of the pressure changes is:

(4) $$\Delta \alpha_{xd}^{}=\kappa \left(\chi_{w}^{x}-\chi_{w}^{d}\right),\;\kappa =\frac{p^{o}}{\tau_{w}}(1-\sum_{j}f_{jw}\chi_{0j}^{o}).$$

The parameter κ (Pa/s) in Equation (4) depends on the outer reference gas composition ${\left\{\chi_{0j}^{o}\right\}}_{j=1,2\ldots }$ and the specific cell constant of water vapor $\tau_{w}={\left(gP_{w}\right)}^{-1}$. Equation (4) can be applied without having to known the exact composition of the outer reference gas by comparing two pairs of simultaneously operated identical chambers. Due to identical κ of the pairs the following equality holds:

(5) $$\varphi =\Delta \alpha_{xd}/\Delta \alpha_{sd},$$

where *s* indicates a reference states of water-saturated air. Assuming linearity between water vapor concentration and pressure change, the unknown mole fraction $\chi_{w}^{x}$ follows for known quantities $\chi_{w}^{s}$ and $\chi_{w}^{d}$ with:

(6) $$\chi_{w}^{x}=\varphi \left(\chi_{w}^{s}-\chi_{w}^{d}\right)+\chi_{w}^{d}$$

**Figure 1:**
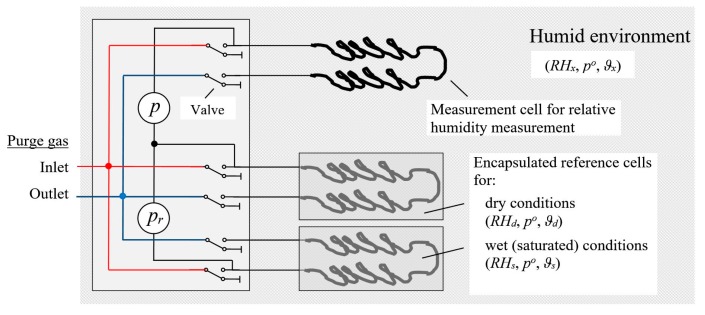
A reference-based humidity sensor consists of a measurement cell exposed to the humid environment at pressure $p^{o}$, temperature $\vartheta_{x}$ and with an unknown relative humidity $RH_{x}$, and two encapsulated reference cells conserving a dryer (*RH_d_*) and a wet (*RH_s_*) reference state of relative humidities for the cell temperatures $\vartheta_{d},\vartheta_{s}$. The flow of a purge gas through the cells is controlled by valves. Pressure sensors *p* and *p_r_* enable the measurement of the differential pressures $\Delta p_{xd}$ and $\Delta p_{sd}$ between the chambers.

The use of the same dry air reference state in Equations (5) and (6) allows one to advantageously reduce the measuring system to three measuring chambers according to Figure 1: Pressure differences $\Delta p_{xd}$ and $\Delta p_{sd}$ between the air of interest and the dryer air reference and between the moist and dryer air references, which are measured with the pressure sensors p and $p_{r}$, suffice to determine $\Delta \alpha_{xd}$ and $\Delta \alpha_{sd}$.

From the structure of Equation (6) follows that the mole fractions used here can be replaced by any variable that is directly proportional to the mole fraction, e.g., by the water vapor pressure. For this case the vapor pressure of water vapor saturation $p_{w}^{s}=p^{o}\chi_{w}^{s}=e_{w}^{\prime }$ is given from the vapor-pressure curve developed already in 1871 by Lord Kelvin [35] and improved later, e.g., in Appendix 4.B of [36] for moist air over a pure water phase (−45 to 60 °C):

(7) $$e_{w}^{\prime }=a\cdot \exp\;[f\cdot \vartheta /(h+\vartheta )]$$

with $a=6.112\;\mathrm{hPa}\cdot (1.0016+3.15\cdot 10^{-6}\;{\mathrm{hPa}}^{-1}p^{0}-0.074\;\mathrm{hPa}/p^{0})$, f= 17.62 °C^−1^ and h= 243.12 °C.

If all measurement cells and their surroundings have the same temperature $\vartheta_{x}$ (°C), Equation (6) can be rearranged with respect to the relative humidity $RH_{d}^{}=\chi_{w}^{d}/\chi_{w}^{s}\cdot 100\%$ of the dryer reference state to give the relative humidity $RH_{x}$:

(8) $$RH_{x}=\varphi \left(RH_{s}^{}-RH_{d}^{}\right)+RH_{d}^{}.$$

For a measurement with respect to a dry reference cell with $RH_{d}^{}\approx 0$, Equation (8) simplifies to $RH_{x}=\varphi \cdot 100\%$, i.e., the parameter φ ranges between 0 and 1 ($RH_{s}=100\%$).

For compensating the influence of temperature difference $\vartheta_{s}-\vartheta_{x}$ of the measurement cells we consider the temperature-dependent relative difference of saturated vapor pressures $\left[e_{w}^{\prime }(\vartheta_{s})-e_{w}^{\prime }(\vartheta_{x})]/e_{w}^{\prime }(\vartheta_{x}\right)$. To this, we can apply Equation (7) on $e_{w}^{\prime }(\vartheta_{s})$ and $e_{w}^{\prime }(\vartheta_{x})$, which results in $\exp[f\cdot (\vartheta_{s}/(h+\vartheta_{s})-\vartheta_{x}/(h+\vartheta_{x}))]$. For $h+\vartheta_{s}\approx h+\vartheta_{x}$ the exponent can be approximated by $f\cdot (\vartheta_{s}-\vartheta_{x})/(h+\vartheta_{x})$. Because $h>>\vartheta_{x,s}$, small differences between the cell-temperatures enable a series expansion of the exponential function (Equation 4.2.19, p. 105 in [37]). Keeping only the first two terms results in the coefficient $\varepsilon_{s}$:

(9) $$\varepsilon_{s}=1+f\frac{\vartheta_{s}-\vartheta_{x}}{h+\vartheta_{x}}$$

and analogously for $\varepsilon_{d}$ when considering a temperature difference $\vartheta_{d}-\vartheta_{x}$ to the dryer reference cell. Combining Equations (8) and (9) results in the equation of a MHS:

(10) $$RH_{x}=\varphi \left(\varepsilon_{s}RH_{s}^{}-\varepsilon_{d}RH_{d}^{}\right)+\varepsilon_{d}RH_{d}^{}.$$

If all three measurement cells have the same environmental temperature, Equation (10) simplifies to Equation (8), i.e., the measurement will be temperature-independent.

The differential pressure changes of $\Delta \alpha_{xd}$ and $\Delta \alpha_{sd}$ in Equation (5) ideally need to be determined within the time $t_{lin}$ after the measurement step has been initiated. Presuming a pressure build-up with $1-\exp(-t/\tau_{w})$ in first approximation, [27], the time $t_{lin}$ can be estimated to $c\cdot \tau_{w}$, e.g., with *c* = 0.044 for an approximation error smaller than 10^−3^. Depending on the geometry factor g of a measurement cell, the high permeability of water vapor can cause $t_{lin}$ to be small, which can restrict the time for pressure analysis. Short measurement intervals will lead to larger measurement uncertainties. Hence, the optimum time range for pressure measurement may be larger than $t_{lin}$. Our experiments show that the pressure build-up between the measurement cells scales with $t^{1/2}$ for times $t<10\;t_{lin}$. Thus, a differential pressure evolution of the form

(11) $$v+u\cdot {\left(t/t_{lin}\right)}^{1/2}$$

can be expected with fitting parameters v and u. Taking the time derivative gives for $t\to t_{lin}$ the approximation $u{\left(2\;t_{lin}\right)}^{-1}$. Hence, parameter φ according to Equation (5) is given by the ratio of $u_{xd}$ and $u_{sd}$ fitted to the differential pressure curves $\Delta p_{xd}$ and $\Delta p_{sd}$. 

We can develop correction procedures for unavoidable minor differences between the construction/properties of the three cells of MHS and the response characteristic of the pressure sensors. When exposing the measurement cell (the top cell in Figure 1) and the dry reference cells (the middle cell in Figure 1) to the same air, the readings for the top pressure sensor in Figure 1 (denoted p) should result in a value of zero for the pressure change between the two cells. If it does not, an offset $off_{xd}$ can simply be determined and used to correct $u_{xd}$. Exposing both reference cells (the middle and bottom cells in Figure 1) to the same air gives analogously the offset determined from the record of the second pressure sensor (labeled $p_{r}$ in Figure 1), which can then be used to correct $u_{sd}$. If the measurement cell is exposed to the same, vapor-saturated air as the wet reference cell, the ratio of $u_{xd}$ over $u_{sd}$ should be equal to one when the offset-corrected values are used. Any deviations can be used to correct the ratio φ in Equation (5).

The sensor theory developed above is based on the assumption of linearity between the measured pressure changes and the external water vapor pressure. To test the validity of this assumption, a MHS must be run without calibration.

### 2.2. Experimental Setup

**Figure 2:**
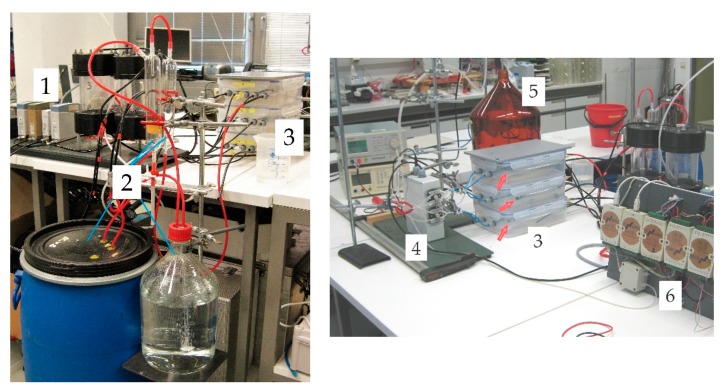
Experimental set-up: MFC’s (1) and a simple humidity generator (2) were used to control the flows and the relative humidity within the stacked containers (3) and of the purge gas for the MHS. The actuating unit of the MHS (4) was connected to the gas sensitive silicone tubes within the containers via gas-tight tubes (blue). Each container was equipped with a reference sensor EE60 (red arrows). A glass tube dipped into a water-filled bottle (5) was used to define the purge gas pressure. For data acquisition and control an ADLink ND-6000 series network (6) was run via PC.

We prepared a humidity sensor consisting of three tubular measurement cells equal in size using silicone tubing (Silicone Peroxide, Fisher Bioblock Scientific, Illkirch, France). The tube ($R_{i}$ = 1.6 mm, $R_{o}$ = 2.4 mm) was cut into pieces of 1 m length. For individual adjustment of test conditions these tubes were placed separately on grids in three sealed plastic containers 295 × 230 × 84 mm^3^ shown in Figure 2 (tag 3). For pressure equilibration ($p^{o}=p_{air}$) with the outer air pressure $p_{air}$, each container was equipped with a mini syringe filter (pore size 0.2 µm, filter diameter 4 mm). One of the three containers was designated the test container (represented by the top cell in Figure 1) and was modified to allow the adjustment of the relative humidity level within it. One of the remaining containers was designed to have relatively dry air in it (dry container), the other one to hold air saturated with water vapor (wet container). These reference containers are represented by the middle and the bottom cell in Figure 1, respectively. Gas-tight tubes (about 20 cm long, $R_{i}$ = 1 mm) were used to connect the gas-selective silicone tubes in the containers to external pinch valves (108P8NO12-01B, Bio-Chem Fluidics, Inc., Boonton, NJ, USA). In order to control the cyclic measurement of the MHS (see Section 2.1), an actuator unit was designed containing the microprocessor controlled pinch valves (label 4, Figure 2). In addition, the microprocessor (ATMEGA328P-PU, Atmel Corp., San Jose, CA, USA) was used for registration of digital pressure data, conversion into pressure units, offset-correction, and averaging, generation of the time stamps for each measured/averaged pressure, and transmitting the data via a TTL/USB-converter to a computer. Self-written C codes were used to run the microprocessor and for data storage on the PC. For pressure measurement pressure sensors of type AMS 5812-0000-D-B were used (pressure range ± 5.17 hPa, precision 2% of full scale within a temperature range of −25 to 85 °C, Amsys GmbH & Co, Mainz, Germany). The time span for the conditioning step was set to 110 s, and that for the measurement step to 4 s. The conditioning step caused a formation of a pressure gradient within the tubular measurement chambers, which equilibrated after closing the measurement chambers at the start of the measurement. To minimize the influence of this relaxation process on the measurement signal, an offset time of 100 ms was allowed between the closing of measurement chambers and starting the pressure registration. Dry air from a compressor was used as purge gas. Its flow through the measurement chambers was pre-adjusted by mass flow controllers (MFC 8710, range 0–5 L/min for air, Bürkert Fluid Control Systems, Ingelfingen, Germany) (label 1, Figure 2) and controlled by a pressure buildup of 20 hPa upstream of the pinch valves. For pressure adjustment a glass tube dipped into a water-filled bottle (label 5, Figure 2) was used.

Water vapor saturation was established within the wet container by a layer of distilled water on the container bottom well below the grid that supported the MHS tube. To adjust the humidity levels in the other two containers we fitted diffusive gas exchangers below the grids within these containers that were made from silicone tubes (length: 4.5 m, inner diameter: 9 mm, outer diameter: 11 mm) with walls that were highly permeable to water vapor (see Table 1). The tube within the dry container was upstream connected to a mass flow controller (MFC 8710, range 0–5 L/min, Bürkert Fluid Control Systems) (label 1, Figure 2) fed with dry compressor air. The tube in the test container was connected to a simple humidity generator (label 2, Figure 2). The first component of the humidity generator consisted of water-filled vessels through which air bubbled up to saturate it with water vapor for ambient room temperature. The maximum relative humidity that could be achieved in the test container was approx. 80%. In the second component, the humidity of the air entering the test container was controlled by mixing this water-saturated air with dry air from the compressor. Each of the two air flows was controlled by a mass flow controller (MFC 8710, range 0–5 L/min, Bürkert Fluid Control Systems) (label 1, Figure 2) that allowed the mixing ratio to be regulated to achieve any desired relative humidity of the air entering the test container. The desired air mixtures were fed into the diffuser tubes that were laid out in the dry and the test container. Water vapor diffused through the tube walls until the relative humidity in the containers reached that inside the diffuser tubes. This process avoided air flow and pressure oscillation inside the containers that could disturb the measurements and the entry of contaminants, e.g., oil, within the compressor air. Moreover, this diffusive equilibration of humidity within the containers guaranties the comparatively fast adjustment of the MHS to a slowly changing *RH* value.

The three containers were placed on top of each other and wrapped in several layers of cloth to approximate temperature equilibration. Nevertheless, small temperature differences existed. These were used to test the performance of Equation (10).

In each container we installed a sensor (EE60, E + E Elektronik, Engerwitzdorf, Austria) that measured both the humidity and the temperature to serve as an independent reference (denoted EE below). For data acquisition and control of reference sensors and MFCs an ADLink ND-6000 series network (ADLINK Technology GmbH, Mannheim, Germany) (label 6, Figure 2) was run via PC. The EE’s measurement range for humidity ranged from zero to full saturation with a precision of 2.5% of the measured value. The temperature range was between −40 and 60 °C, with a precision of 0.3 °C. Instead of using the factory calibration curve, we calibrated the temperature and RH readings of the EE sensors against a certified resistive-electrolytic portable meter (XP201, Lufft Mess- und Regeltechnik GmbH, Fellbach, Germany). The XP201’s humidity range was also between zero and saturation, with a precision of 0.8% RH (equivalent to two standard deviations for 15–30 °C temperature range). This precision is superimposed to the accuracy of the sensor’s reference standard of 0.3 to 0.7% RH (calibration mark: 1867, D-K-15202-01-00, 2019-01). The temperature range was between 0 and 70 °C with a precision of 0.15 °C.

**Figure 3:**
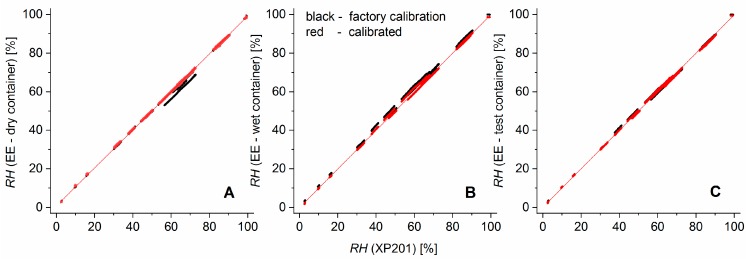
Factory calibrated (black dots) and recalibrated (red dots) relative humidity (RH) of EE sensors installed in the dry (**A**), wet (**B**), and test container (**C**). The red lines are the sensor characteristics based on our own calibration.

Figure 3 illustrates the improvement of the calibration over the factory calibration. The residuals of the EE humidity measurements versus calibration curve had a standard deviation of 0.4% (EE in the dry container), 0.6% (wet container), and 0.55% (test container). For the temperature, the standard deviations of the residuals were 0.019 °C (dry container), 0.013 °C (wet container), and 0.014 °C (test container).

The shift from the factory calibration (black dots) to the user-calibrated sensors (red dots, final calibration curve: red line) was achieved in a two-step calibration procedure: First the temperature dependence of the *RH* signal was recalibrated over a temperature range from 10 to 35 °C by applying a quadratic regression polynomial on the *RH* differences from EE sensors and the XP201 with respect to the previously calibrated EE sensor temperature. This additive correction was sensor-specific but behaved nearly independently of the relative humidity. It shifted measurement values parallel to the EE—axes of the sensor characteristics and, thereby, reduced the artefact of having parallel bands of relative humidity values (Figure 3). In essence, the need for this elaborate recalibration arose from the very high sensitivity of the relative humidity reported by the sensors to the temperature. In the second step, a quadratic regression polynomial was fitted to calibrate the temperature-corrected EE humidity sensors against various humidity levels in a range from 3 to 100% for ambient temperature.

### 2.3. Experimental

Every 10 s the EE sensors, provided near-continuous reference readings for the relative humidities and the temperatures. Each reading was the average of 50 measurements acquired in the 10 s time interval. The measured reference values were converted to their final values using the calibration relationships that were determined as described in Section 2.2.

Cyclic conditioning and measurement steps were carried out continuously by the MHS. Thereby, 100 pressure readings from the pressure sensors *p* and *p_r_* were obtained every 120 s during the 4 s interval of a measurement step. From these pressure readings $u_{xd}$ and $u_{sd}$ were estimated and $RH_{x}$ was computed according to Equation (10). To examine whether the assumption of saturated water vapor in the wet container can be safely used (assuming $RH_{s}^{}=100\%$ in Equation (10)), MHS-derived relative humidities were also calculated using the independently measured relative humidity from the EE sensor in the wet container.

During a three-day initial period, the dry and the test container were equilibrated with dry compressor air (RH ≈ 4%). After this time, both the dry and the test container had dry air in them. We used the opportunity to determine an offset $off_{xd}=$ 0.0117 ± 0.0010 hPa/s between the measurement cells in the test container and the dry container based on a time series of several hours. Then, the experiment for the measurement of various humidity levels was started. The wet container was kept closed during the duration of the experiment to allow the air to be in a vapor pressure equilibrium with the water layer at the container bottom. The humidity levels in the test container were established by diffusive equilibration as described above with air of the desired relative humidity for at least 24 h. In a period of 8 days, 7 humidity levels (ranging from 4 to ca. 80%) were established. The final saturation humidity level was achieved by pouring distilled water into the test container to create conditions similar to that in the wet container, after rearranging and testing the set-up to allow the direct addition of water to that container. Due to the rearranging this final step started 17 days later. DASYLab 10.0 (dasylab.com) was used to control the MFCs and for reference data acquisition, Mathematica 11.1 (Wolfram Research, Champaign, IL, USA) for overall data analysis.

## 3. Results

**Figure 4:**
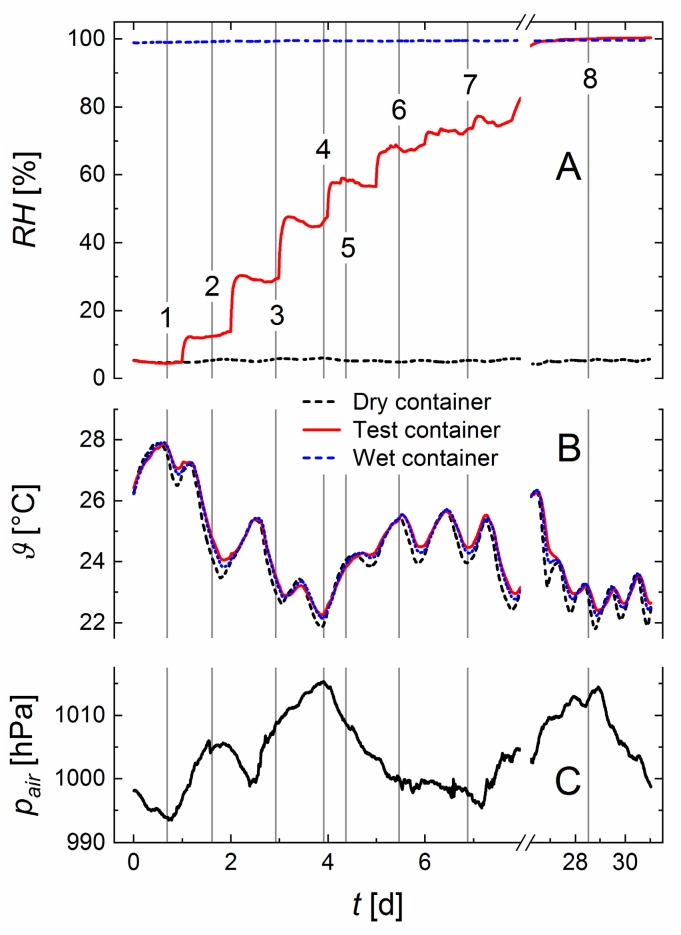
(**A**) Relative humidities RH and (**B**) temperatures ϑ in the three containers. The air pressure $p_{air}$ in the laboratory is shown in **C**. Numbered gray lines refer to selected data sets for which the measurement with a MHS is explicitly demonstrated below.

Figure 4 shows the evolution (A) of relative humidities and (B) temperatures in all containers. The laboratory air pressure $p_{air}$ is shown in C. Each measurement series consist of 9220 measurements.

The time-axis divides the data in two experimental sections: During the first eight days the relative humidity was varied stepwise from 4 to 80%. Between day 26 and 31 the air in the test container was saturated with water vapor. Despite a diurnal temperature variation in the laboratory and a change of the atmospheric air pressure of 23 hPa in the experimental period, the relative humidities in the reference containers were stable.

Figure 4 shows that the stepwise mixing ratio given by the MFC’s did not lead to constant relative humidity levels in the test container. Due to the diffusive adjustment of relative humidity (see Section 2.2) the time intervals for acquiring a relative humidity measurement (measurement time 4 s, setting time in the order of the cell constant $\tau_{w}=$ 19 s) were short compared to the rate of change in $RH_{x}$. The same is true for the diurnal temperature changes. So these temporal variations did not compromise the test of the MHS. To demonstrate the operation of the new sensor in more detail, data sets were collected at the positions of the numbered gray lines in Figure 4.

**Figure 5:**
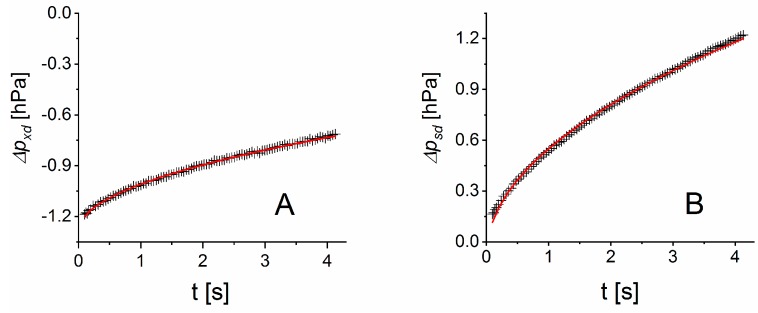
Measured (black crosses, taken from data set No. 4 in Figure 4) and fitted (red lines) pressure differences with the dry measurement cell of the measurement cells in the test container (**A**) for a relative humidity of 46.0% and in the wet container (**B**). Note that the vertical scales are different.

Figure 5 shows the fitted differential pressures for position 4 in Figure 4. The geometry factor of the measurement cells was $g=1.93\times 10^{6}$ m^−2^, leading to the cell constant of $\tau_{w}=19$ s. This resulted in a time $t_{lin}\approx 0.8$ s. Hence, as clearly shown in Figure 5, we expect a nonlinear pressure evolution. Thus, the pressure changes $\Delta \alpha_{xd}$ and $\Delta \alpha_{sd}$ have to be approximated using the fitted model (red lines) according to Equation (11). Figure 5 shows that Equation (11) represented the data with sufficient accuracy.

The differential pressures were measured with respect to the pressure in the membrane tube that was placed in the dry container (Figure 1). This container had a relative humidity of $RH_{d}=$ 6.1%. By fitting these differential pressure curves the parameters $u_{xd}=$ 0.250 hPa/s and $u_{sd}=$ 0.571 hPa/s were obtained. A relative humidity value $RH_{x}=$ 47.1% follows from Equation (8). The offset-reduced pressure change $u_{xd}-off_{xd}$ (Section 2.3) results in a relative humidity estimate of $RH_{x}=$ 45.2%. The deviations of these estimates from the EE-measured adjusted relative humidity RH in the test container of 46.0% are within the precision of 1.1% RH of the EE sensor (two standard deviations). The membrane dependent sensitivity follows from $u_{sd}{\left(RH_{s}-RH_{d}\right)}^{-1}$ with $6\times 10^{-3}$ hPa (s ⋅ % RH)^−1^.

**Figure 6:**
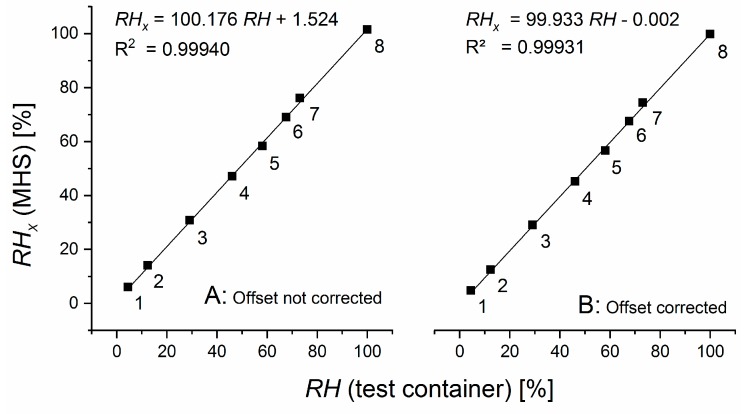
Relative humidity $RH_{x}$ measured with the MHS without (**A**) and with (**B**) considering the correction of an offset. Regression lines and correlation coefficients demonstrate a high correspondence with the adjusted relative humidity RH analyzed simultaneously with the EE sensor.

Figure 6 shows the relative humidity $RH_{x}$ calculated from the MHS data for all marked positions in Figure 4. Both calculations without (A) and with (B) considering the offset correction show a good match with the relative humidity RH measured independently in the test container. Regression coefficients near 1 confirm MHS was functioning well. The offset correction in (B) brought the intercept down to zero within four significant digits, and marginally improved the approximation of a slope of one, which was already very accurate without the offset correction.

Figure 6 demonstrates that the equations that derive the relative humidity from the observed pressure differences in the MHS performed excellently. Specifically, it proves that the equality in Equation (5) holds over the full range of relative humidity examined. The high correlation coefficients confirm the validity of the linearity assumption of the membrane-based measurement method for water vapor pressure.

**Figure 7:**
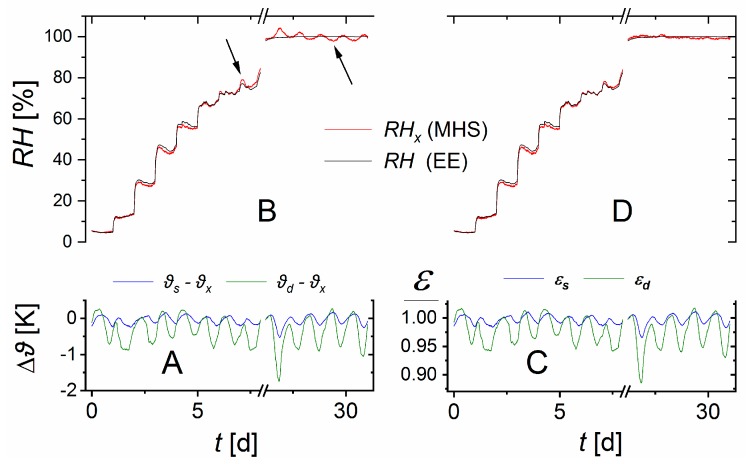
(**A**) Temperature differences Δ*ϑ* between the wet (*ϑ_s_*), the dry (*ϑ_d_*), and the test container (*ϑ_x_*). (**B**) These differences caused variations of the measured relative humidity *RH_x_* around the expected *RH* in the test container. The arrows point to clear deviations from the independently measured values. (**C**) The coefficients *ε_d_*, *ε_s_* calculated by Equation (9). (**D**) Using these coefficients in Equation (10) improved the relative humidity calculated from MHS data.

As shown in Figure 4 and Figure 7A, temperature differences between the containers occurred. Ignoring these by using Equation (8) causes fluctuations of the measured relative humidity $RH_{x}$ around the expected relative humidity *RH*. These fluctuations increased with increasing relative humidity (see arrows in Figure 7B). Using Equation (10) instead, with the corrections (Figure 7C) based on the vapor pressure curve removed most of these fluctuations (Figure 7D).

**Figure 8:**
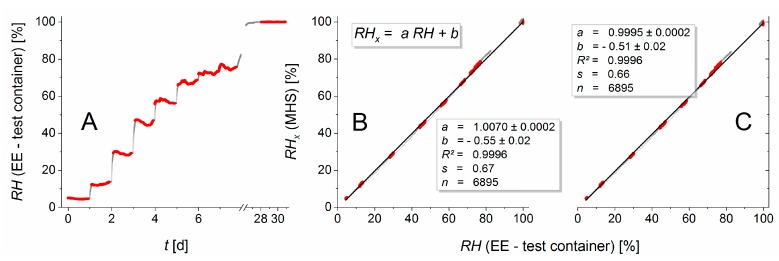
(**A**) The relative humidity in the test container, measured with the EE sensor. The complete data set is displayed in grey. Highlighted in red are the plateau regions for the regression analysis in (**B**,**C**). (**B**) Membrane based humidity measurements that assumed water vapor saturation in the wet container. (**C**) Like **B**, but instead of assuming vapor saturation, the measurements of the EE sensor were used in the wet container. The number of data points (*n*) in panels B and C refers to the highlighted data points for the regression analysis. The regression coefficient *a* and the offset *b* are displayed with their standard errors. *R*^2^ denotes the squared correlation coefficient and *s* is the standard dispersion of residuals.

Figure 8 shows (A) the evolution of relative humidity in the test container determined with the calibrated EE sensor in comparison with the measurement results of the membrane based humidity sensor (B, C). Our experiments, however, showed that the EE sensor reacted much more slowly to a change in humidity than the membrane based sensor. In order to ensure a spatially adjusted water vapor equilibrium and the adjustment of both sensor types, plateau regions were defined within which comparatively slow changes in humidity occurred.

These plateau regions (red highlighted in Figure 8, number of data points n = 6895) were used as the basis for a regression analysis. The entire data set (n = 9220) is displayed in gray. Membrane based humidity measurement was performed assuming (B) water vapor saturation in the wet container, i.e., *RH_s_* ≡ 100% or using the measurement values of the calibrated EE sensor in this container (C). Both measurement variants demonstrated a high correspondence of the MHS measurement in the test container with the EE reference measurement. Both the regression coefficients (*a*) and the squared correlation coefficients *R*^2^ are close to 1. The offsets (*b*) and the standard dispersion of residuals (*s*) are smaller than 0.7%. This measurement results are within the confidence range of the EE sensor.

The difference between the outcomes reported in Figure 8B,C is insignificant. No separate measurement of the relative humidity for saturation in the wet container is necessary. Therefore, Equation (10) can generally be written as follows

(12) $$RH_{x}=\varphi \left(\varepsilon_{s}\cdot 100-\varepsilon_{d}RH_{d}^{}\right)+\varepsilon_{d}RH_{d}^{}$$

and only the temperature differences between the measurement cells and the relative humidity to which the dry measurement cell is exposed need to be known.

## 4. Discussion and Conclusions

Various measurement principles are in use for the measurement of the relative humidity of gas mixtures. Typically, the raw signals of the sensor are highly sensitive to the temperature of the gas mixture and therefore require accurate temperature measurements. This is illustrated by the elaborate recalibration we were forced to perform for the EE sensors (Section 2.2). We introduce a new measurement principle that is based on the water vapor pressure-driven diffusion. It results in a direct response to the relative humidity, and is not sensitive to the temperature of the gas mixture. An indirect dependence on the temperature results only from the (independent) determination of the relative humidity for the dry reference cell.

Furthermore, our results prove the applicability of our steady state-based theory on an expanded time interval for obtaining the measurement signal. This significantly expands the possible time interval for an optimal measurement.

The method requires linearity between the measured pressure changes and the water vapor pressure in the gas mixture. This paper therefore elaborately tested if this was the case for air as the gas mixture. The high correlation coefficients shown in Figure 6 and Figure 8 convincingly confirm the validity of the linearity assumption. This constitutes proof of principle of the MHS, and thus opens the door to future applications without the necessity of very accurate additional measurements of temperature, which are required by existing sensors.

For applications in soil, it is worthwhile to note that effects of direct solar radiation, wind, etc. are absent. Daily temperature fluctuations are often damped out effectively in only a few decimeters depth or less in humid soils because of the high heat capacity of the soil water. In dry soils, the daily temperature signal is likely to be a factor for a buried sensor. The water vapor pressure should be expected to be non-uniform because of partial shading of the soil surface by the crop, non-uniform soil water distribution, and soil heterogeneity. For a uniform environmental temperature around the measurement cells of an MHS, all cells have the same temperature, and thus no temperature information is needed for determining relative humidity. Due to the proven linearity, this should also be valid for locally varying environmental temperatures along the MHS that result in locally different water vapor pressures. This sets our technique apart from existing techniques.

Moreover, our theory enables considering slight differences of the mean temperatures between the measurement and reference cells. The necessary information about the mean temperature in the surroundings of the particular cell can be gathered from the purge gas temperature. Thus, also in this case an MHS is capable to accurately measure the relative humidity.

We found that the MHS functioned very well during the experimentally encountered temperature range between 22 and 28 °C. Soil temperatures in hot, arid regions can reach 80 °C as measured in a dry Tanzanian soil [13]. For such extreme applications, separate, dedicated testing of the performance in a wider temperature range is needed. In Figure 8, slight deviations between the measurement values and the regression lines are visible, but we do not yet know which sensor(s) or conditions caused this. If such deviations increase substantially with increasing temperature ranges, it might be necessary to resort to reference measurements of higher accuracy.

As discussed in [30], C-turnover in soil can result in elevated concentrations of CO_2_ of up to 10%, which can according to the comparatively high permeability of CO_2_ (Table 1) influences the measurement. But in these cases water must be abundant, i.e., *RH* ≡ 100%. In dry soil, turnover processes are limited by water demand. The increased air-filled porosity in a drier soil results in an enhanced gas exchange with the atmosphere. Both processes will lower the difference of the soil gas composition with respect to air. Nevertheless, further investigations will be appropriate with respect to influences due to varying gas composition on the measurement and for compensating techniques, e.g., in analogy to those developed in [30].

For our investigations silicone was selected as a water vapor sensitive membrane. The gas permeability coefficients for commercially available silicone rubber materials are generally not provided by manufacturers. They may actually vary depending on the production process and the composition, e.g., the content of fillers. A study on the permeability of gases and water vapor in a silicone membrane in [34] enables a consistent estimate of gas selectivity coefficients *f_jw_* with respect to water vapor as used in Equation (4). Table 1 shows gas selectivity coefficients for gas components that are present in air in sufficient concentrations to contribute to a change in pressure in the measurement chamber. The table clearly shows why silicone is well suited as a membrane material: both its permeability to and its selectivity for water molecules exceeds that for the other components of air. Moreover, silicone is inexpensive, available in a wide range of dimensions, and can be manufactured with small tolerances in various formulations. The material is durable under prevalent environmental conditions over years. These properties indicate that silicone is a suitable membrane material for long-term monitoring of the relative humidity in dry soils. Beyond that the availability of various further membrane materials, e.g., collected in [38,39], allows for tailored MHS construction for sensor applications/environments for which silicone is not suited without changing the measurement principle.

Using silicone as a membrane, we proved the linearity of the measurement signal over the full range of relative humidity examined, approx. 4 to 100% for temperatures between 22 to 28 °C and an air pressure range of 993 to 1015 hPa. As explained in Section 2.1, this linearity is the basis of the sensor theory that we developed to determine an unknown relative humidity from simultaneously measured responses to known levels of relative humidity. These internal references eliminate the need for external calibration.

An important and highly desirable feature that arises from the linearity is the possibility to use measurements with a single tubular MHS that samples a heterogeneous environment to find the arithmetic mean of a locally varying relative humidity. This opens the possibility to use such a sensor to derive representative relative humidity readings for dynamic natural systems such as soils, where heterogeneity exists on different scales. The sensor can therefore potentially be used to determine the degree of water stress experienced by a crop and optimize irrigation based on this information. The robustness of the sensor also allows representative humidity measurements that may improve the control of technical infrastructures such as storage silos, greenhouses, etc. Due to the internal reference for water vapor saturation, an MHS could be effectively used for high relative humidity, since commercially available sensors here usually have the highest measurement uncertainties.

## Figures and Tables

**Table 1:** Silicone membrane permeability of gas components of air from Robb [34] (converted ^(^^1)^ to (m^2^/s)) and gas selectivity coefficients $f_{jw}$ with respect to water vapor.

Gas Component j	$P_{j}\times 10^{9}(m^{2}/s)$	$f_{jw}$
H_2_O	27.0	1
N_2_	0.21	0.008
O_2_	0.45	0.017
Ar	0.45	0.017
CO_2_	2.44	0.090

^(1)^ Conversion factor: 75 × 10^−4^ cmHg.
